# Experiential Relationship between Malaria Parasite Density and Some Haematological Parameters in Malaria Infected Male Subjects in Port Harcourt, Nigeria

**DOI:** 10.5539/gjhs.v4n4p139

**Published:** 2012-06-15

**Authors:** Eze Evelyn M., F. C. Ezeiruaku, D. C. Ukaji

**Affiliations:** 1 Department of Haematology and Transfusion Science, Madonna University, Elele, Nigeria; 2 Department of Medical Laboratory Science, Niger Delta University, Yenegoa, Nigeria; 3 Department of Microbiology, Madonna University, Elele, Nigeria

**Keywords:** parasite density, malaria, *Plasmodium*, neutrophil, eosinophil, lymphocyte, haemoglobin, haematocrit, platelets

## Abstract

This study examined the experiential relationship between the parasite density and haematological parameters in male patients with *Plasmodium falciparum* infection in Port Harcourt, Nigeria reporting to malaria clinics. A total of one hundred and thirty-six (136) male patients were recruited. QBC haematological analysis, QBC malaria parasite specie identification and quantification and thin blood film for differential leucocytes count was used. The mean values of the haematological parameters in each quartile of parasite densities were determined using Microsoft Excel statistical package. Regression analysis was employed to model the experiential relationship between parasite density and haematological parameters. All regression relationships were tested and the relationship with the highest coefficient of determination (R^2^) was accepted as the valid relationship. The relationships tested included linear, polynomial, exponential, logarithmic and power relationships. The X- axis of the regression graphs stand for the parasite density while Y-axis stands for the respective haematological parameters Neutrophil count had a negative exponential relationship with the parasite density and is related to the parasite density by a polynomial equation model: y_nm_ = -7E-07x^2^ - 0.0003x + 56.685. The coefficient of determination (R^2^) was 0.6140. This means that the rate of change of the parasitemia will depend on the initial value of the neutrophil. As the neutrophil increases, the parasitemia will tend to decrease in a double, triple and quadruple manner. The relationship between lymphocyte count, monocyte count and eosinophil count and parasite density was logarithmic and expressed by the following linear equation models: y_lm_ = -2.371ln(x) + 37.296, y_mm_ = 0.6965ln(x) + 5.7692 and y_em_ = 0.9334ln(x) + 4.1718 in the same order. Their respective high coefficients of determination (R^2^) were 0.8027, 0.8867 and 0.9553. This logarithmic relationship means that each doubling of monocyte count and eosinophil count will cause the same amount of increase in parasitemia whereas each doubling of lymphocyte count will cause the same amount of decrease in parasitemia. The best fitting regression model for total white cell count (WBC), haemoglobin concentration, packed cell volume (PCV)(haematocrit) and mean cell haemoglobin concentration (MCHC) and parasite density was a linear model and expressed by the following linear equation models: y_WBCm_ = 1.2314x + 8533.8, y_Hbm_ = -0.0014x + 13.004, y_PCVm_ = -0.0046x + 41.443 and y_MCHCm_ = -0.0008x + 32.336. Their respective coefficients of determination are 0.7397, 0.6248, 0.9758 and 0.8584. This linear relationship means that as the parasite density is increasing that there is a corresponding decrease in haemoglobin concentration, PCV and MCHC and a corresponding increase in total white cell count. The best fitting regression model between platelet count and parasite density is a power model with a very high coefficient of determination (R^2^=0.9938) and expressed by: y_Pltm_ = 278047x^-0.122^. These equation models could be very useful in areas where there may not be functional microscopes or competent microscopists and in medical emergencies.

## 1. Introduction

Malaria infection is caused by invasion of red blood cells with protozoan parasites of the genus *Plasmodium*. The female anopheles mosquito is the carrier of the parasite and transmits it to man by next blood meal. Malaria is widespread in tropical and subtropical regions because of the significant amounts of rainfall and consistent high temperatures and high humidity, along with stagnant waters which provide mosquitoes the environment needed for continuous breeding (Prothero & Mansell, 1999).

The bite of mosquito that carries the plasmodium leads to the presence of the parasite in red blood cells, causing symptoms that typically include fever and headache, in severe cases progressing to coma and death. The four *Plasmodium* species that infect humans are *P. falciparum*, *P. vivax P. ovale* and *P. malariae*. Occasional infections with monkey malaria parasite, such as *P. knowlesi*, also occur (Roberts et al., 2002).

Malaria infection is a major public health problem in tropical areas, and it is estimated that the disease is responsible for 1 to 3 million deaths and 300-500 million infections annually worldwide (Desowitz, 1993; Philips & Nicky, 2010). Malaria is holoendemic in Nigeria with *Plasmodium falciparum* as the dominant strain (Lesi & Adenuga, 1996). Malaria parasitemia has been reported to have effects on some haematological parameters in many parts of the world (Perrin et al., 1982; Abdalla, 1988; Rojanasthien, Surakamolleart, Boonpucknavig, & Isarangkura, 1992; Sharma, R.Das, B.Das & P.Das, 1992; Lesi & Adenuga, 1996; Layla et al., 2002; Oseni, Togun, Olowu, & Okoli, 2006). The vast majority of morbidity and mortality from malaria is caused by infection with *P. falciparum*, mostly among children under the age of 5 years living in sub-Saharan Africa (Guerin et al., 2002). The infection with *P. falciparum*, which causes the most severe infections and nearly all malaria-related deaths, has been well documented in areas of high endemicity in Africa (Day & Marsh, 1991).

Haematologic changes, which are the most common complications, play a major role in fatal complications. They include anaemia, cytoadherence of infected red cells, leukocytic changes followed by the induction of cytokines, thrombocytopenia and coagulopathy, particularly disseminated intravascular coagulation (Price et al., 2001).

Malaria parasite identification request is usually accompanied with full blood count for proper diagnosis, treatment and management of malaria infection. There are several methods employed in malaria parasite identification and quantification. Some of these methods include: serological techniques, molecular techniques using polymerase chain reaction (PCR), quantitative buffy coat (QBC) technique and microscopic method using peripheral blood thick and thin film. These techniques are all with some limitations particularly in a resource limited environment like Nigeria.

Several rapid diagnostic tests have been developed which detect malaria parasite antigens in lysed blood using monoclonal antibodies. However, there are currently no known rapid diagnostic tests which can differentiate *P. vivax*, *P. ovale*, or *P. malariae*. These tests are not quantitative and will not provide any information concerning levels of parasitemia. Antigen persistence is also a problem. PCR testing and QBC method offer a rapid, sensitive, and less subjective methods to determine the presence and species of *Plasmodium*. Unfortunately, these malaria tests are expensive and not routinely used in Nigeria.

The gold standard used for malaria parasite diagnosis in Nigeria is the peripheral blood thick and thin film method. This is because it is most economical. It is also reliable, sensitive and specific but the reliability, sensitivity and specificity of this method depend on the efficiency of the microscopist. Unfortunately in many laboratories in Nigeria, particularly in the rural areas, this manpower is lacking.

On the contrary, a good number of laboratory scientist and technicians in Nigeria can easily assess haematological parameters. Many malaria cases particularly children under 5 years come as medical emergency and as such little time is needed to diagnose, treat and save their lives. To reduce malaria-related deaths in future particularly in rura006C Africa, strategies on quick diagnosis and treatment should be adopted. Based on these, this study examined the experiential relationships between some haematological parameters and the parasite density of the malaria infected male subjects in Port Harcourt, Nigeria. These relationships can be used to, given any of the haematological parameter, predict the malaria parasitemia within the shortest time even in the absence of a functional microscope or a competent microscopist. There are sex differences in some haematological parameters hence the need to develop these regression models according to sex.

## 2. Materials and Methods

### 2.1 Study Area

The study was carried out in Port Harcourt located between latitudes 4° 2′ North and 4° 47′ North and longitudes 6° 55′ East and 7° 08′ East has continued to record a high incidence of malaria infection despite the federal government efforts to roll back malaria in Nigeria. The reason is that Port Harcourt is a typical coastal zone located in the Niger Delta of Nigeria. High temperatures and humidity as well as marked wet and dry seasons characterize the climate. The mean annual rainfall is estimated at about 2,405mm while the mean monthly temperature varies between 24°C and 32°C throughout the year (Gobo, 1988). The mean annual temperature for Port Harcourt is 26°C (Gobo, 1988). The mean annual temperature, relative humidity and rainfall of Port Harcourt favour the development of both the parasite as well as the vector.

### 2.2 Study Population

The laboratory study was carried out for a period of six months during which a total of one hundred and thirty- six (136) males were recruited. The inclusion criteria were male out-patients to the participating clinic site within the age of 1- 60 years queried for malaria infection with the presentation of at least one of the following: an oral temperature of 38°C, headache, or a history of fever within the past 72 hours and who must not have commenced any treatments for malaria. Exclusion criteria were male out-patients with pathological conditions outside malaria such as protozoan or helminthes infection, typhoid fever and HIV/AIDS, congenital manifestations such as sickle cell disease and history of allergy. All enrolled patients were interviewed for information on current symptoms and previous malaria episodes and treatments and informed consent obtained from them before blood sample collection.

### 2.3 Blood Sample Collection and Processing

A volume of 2.6 ml of the venous blood sample was drawn into monovette tubes containing the anticoagulant potassium ethylenediamine-tetra acetic acid (EDTA) for QBC haematological analysis, QBC malaria parasite specie identification and quantification and thin blood film for differential leucocytes count. QBC Autoread Plus manufactured by QBC Diagnostic Incorporated, USA provided a diagnostic haematology profile of the following quantitative values from a single tube of blood; packed cell volume (haematocrit), haemoglobin concentration, mean corpuscular haemoglobin concentration, platelet count, white blood cell count, granulocyte count (% and number) and lymphocyte-monocyte count (% and number). Daily quality assurance checks were performed and recorded.

For QBC malaria parasite detection analysis, the centrifuged tube was examined under a fluorescence microscope in the region between the light red blood cells and granulocytes and lymphocytes/monocytes, where the parasites are most abundant. Examination of the centrifuged blood under a fluorescence microscope readily permits the detection of malaria parasite in the infected cells and plasma. Since the parasites contain DNA which takes up the acridine orange stain, they appear as bright specks of light in the dark background of non-fluorescing red cells.

For QBC malaria parasite species identification, at magnification of 600X, all parasites in the red blood cells were easily visualized and their morphologies identified. Species identifications were made based upon the size and shape of the various stages of the parasite and the presence of stippling (i.e. bright red dots) and fimbriation (i.e. ragged ends). Plasmodium parasites are always intracellular, and they demonstrate, if stained correctly, blue cytoplasm with a red chromatin dot.

The parasite densities were obtained by multiplying the average number of parasites in 10 QBC fields by a factor of 10.5 (QBC operator’s manual, 2006).

For a reliable differential leucocytes count, a thin blood film was prepared by a standard manual technique as described by Bain et al. (2008) on a clean grease-free glass slides, allowed to air-dry and fixed in alcohol (methanol) for 2 minutes and then stained with Field’s stain. The differential leucocyte count was carried out by the longitudinal technique.

### 2.4 Data Analysis

The data obtained were grouped into four parasite densities and analyzed using Microsoft Excel statistical package. Regression analysis was employed to model the experiential relationship between parasite density and haematological parameters. All regression relationships were tested and the relationship with the highest coefficient of determination (R^2^) was accepted as the valid relationship. The relationships tested included linear, polynomial, exponential, logarithmic and power relationships. The X- axis of the regression graphs stand for the parasite density while Y-axis stands for the respective haematological parameters.

## 3. Results

As shown in Table 1, heavy infection (3+) represented the highest percentage accounting for 47.79%. The percentage of infected subjects manifesting very heavy infection (4+) was 16.18% while scanty infection (1+) represented the least percentage accounting for 13.97%.

**Table 1:** Distribution of the male infected subjects according to levels of parasitemia

Level of parasitemia	Total	%
scanty infection(+)	19	13.97
moderate infection(2+)	30	22.06
heavy infection (3+)	65	47.79
very heavy infection (4+)	22	16.18
**Total**	**136**	**100.00**

Table 2 depicts the Mean±SD of Haematological Parameters of Males of Different Parasitemia. The value of neutrophil count in males suffering from P.*falciparium* malaria increased gradually from 53.89±9.99 % in scanty infection (1+) to 61.41±4.17 % in very heavy infection (4+). There was increase in neutrophil count with increase in parasitemia. Other haematological parameters in the P.*falciparium* malaria infected subjects include monocyte count which ranged from 6.42±1.87% in scantly infected male subjects to 10.59±2.13% in very heavily infected subjects.

**Table 2:** Mean±SD of haematological parameters of males of different parasitemia

Parameters	1+	2+	3+	4+
N (%)	53.89 ± 9.99	58.37 ± 5.03	58.63 ± 8.65	61.41 ± 4.17
L (%)	34.95 ± 9.58	24.37 ± 4.56	22.11 ± 6.69	17.23 ± 2.99
M (%)	6.42 ± 1.87	8.70 ± 2.61	9.68 ± 3.05	10.59 ± 2.13
E (%)	5.26 ± 1.59	8.57 ± 1.74	9.49 ± 1.91	10.32 ± 1.78
B (%)	0.00 ± 0.00	0.00 ± 0.00	0.00 ± 0.00	0.00 ± 0.00
WBC /mm^3^	6942.11 ± 1868.55	8026.67 ±1904.85	9033.85 ± 2340.76	10231.82 ± 3433.12
Hb (g/dl)	13.67 ± 1.35	13.03 ± 12.44	12.44 ± 1.54	11.02 ± 1.87
PCV (%)	42.00 ± 3.96	40.77 ± 39.09	39.09 ± 4.67	35.15 ± 4.98
MCHC	32.57 ± 1.35	31.93 ± 1.91	31.90 ± 1.57	31.26 ± 1.86
Plt /mm^3^	230526.32 ± 79240.40	167733.33 ± 58997.33	136198.46 ± 35752.55	115545.45 ± 25688.11
Parasite Density P.falciparium(Parasite/μl)	5.05 ± 2.84	50.07 ± 25.53	386.89 ± 262.78	1359.95 ± 309.19

**Figure 1:**
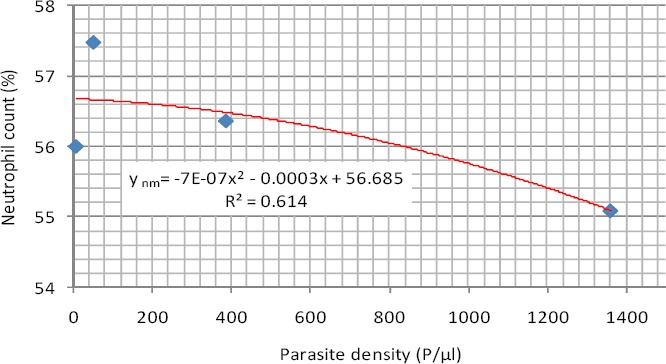
Exponential Relationship between neutrophil count and parasite density (Male)

As shown in Figure 1, the regression analysis of neutrophil count among the malaria infected male subjects had an exponential relationship with the parasite density. The neutrophil count in males (y_nm_) is related to the parasite density x, by the polynomial equation

*y_nm_ = -7E-07x^2^ - 0.0003x + 56.685* (1)

The coefficient of determination (R^2^) was R² = 0.6140.

This negative exponential relationship means that the rate of change of the parasitemia will depend on the initial value of the neutrophil. As the neutrophil increases, the parasitemia will tend to decrease in double, triple and quadruple manner.

**Figure 2:**
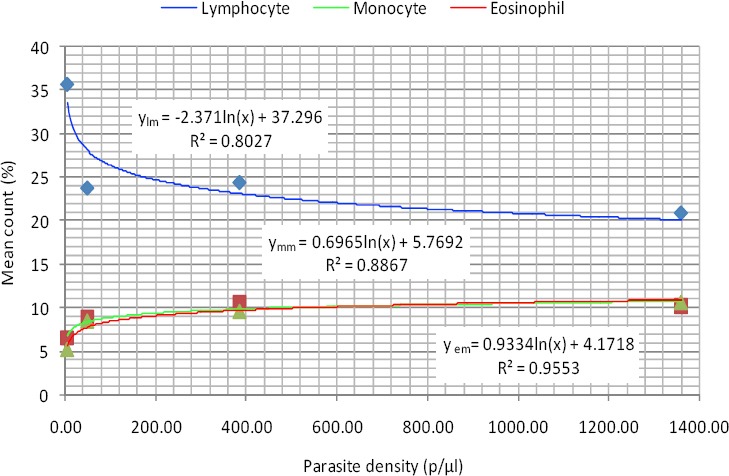
Logarithmic relationship between lymphocyte, monocyte and eosinophil counts and parasite density (Male)

As shown in Figure 2, the relationship between lymphocyte count and parasite density among the same subjects was logarithmic and expressed by the equation. This means that each doubling of lymphocyte count will cause the same amount of decrease in parasitemia.

*y_lm_ = -2.371ln(x) + 37.296* (2)

Where y_lm_ is the lymphocyte count in males. The coefficient of determination was very high (R² = 0.8027).

As also shown in Figure 2, monocyte and eosinophil counts also had logarithmic relationships with parasite density. This means that each doubling of monocyte count and eosinophil count will cause the same amount of increase in parasitemia. These relationships are defined by the equations 3 and 4 for monocyte counts in males (y_mm_) and eosinophil counts in males (y_em_) and. their respective coefficients of determination were 0.8867 and 0.9553.

*y_mm_ = 0.6965ln(x) + 5.7692* (3)

*y_em_ = 0.9334ln(x) + 4.1718* (4)

**Figure 3:**
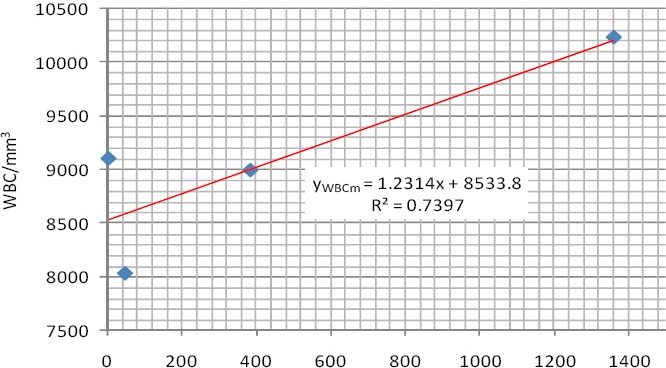
Linear relationship between WBC count and parasite density (Male)

As shown in Figure 3, WBC has a positive linear relation with parasite density and a high value of coefficient of determination (R² = 0.7397). This means that as the parasite density is increasing that there is a corresponding increase in total white cell count.

This relationship is defined by the equation 5 for WBC Count in males (y_WBCm_)

y_WBCm_ = 1.2314x + 8533.8 (5)

**Figure 4:**
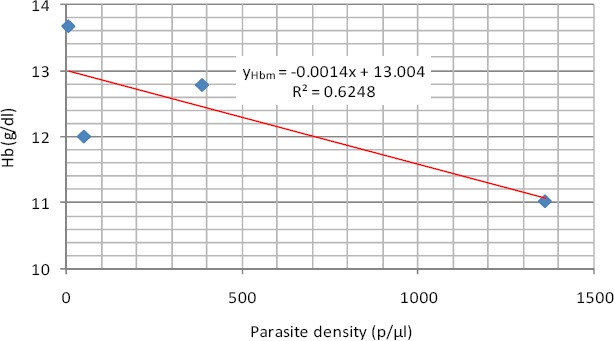
Linear relationship between Hb concentraion and parasite density (Male)

As shown in Figure 4, Hb Concentraion had a negative linear relationship with parasite density with a coefficient of determination of 0.6248. This means that as the parasite density is increasing that there is a corresponding decrease in haemoglobin concentration.

The relationship is expressed in equations 6 where y_Hbm_ is the Hb Concentraion in males

y_Hbm_ = -0.0014x + 13.004 (6)

**Figure 5:**
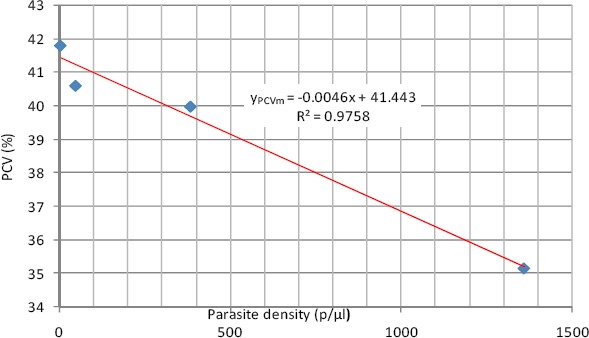
Linear relationship between PCV and parasite density (Male)

As shown in Figure 5, PCV had a negative linear relationship with parasite density with a high coefficient of determination of 0.9758. This means that as the parasite density is increasing that there is a corresponding decrease in PCV.

The relationship is expressed in equations 7 where y_PCVm_ is the PCV in males

y_PCVm_ = -0.0046x + 41.443 (7)

**Figure 6:**
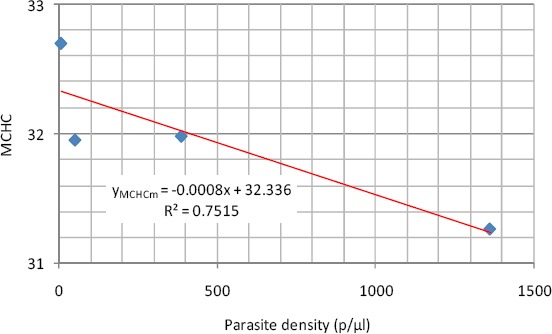
Linear relationship between MCHC and parasite density (Male)

As shown in Figure 6, the regression analysis result of MCHC showed that it has a negative linear relationship with parasite density shown in equation 8. This means that as the parasite density is increasing that there is a corresponding decrease in MCHC.

y_MCHCm_ = -0.0008x + 32.336 (8)

The coefficient of determination was 0.8584 where y_MCHCm_ is the MCHC in males

**Figure 7:**
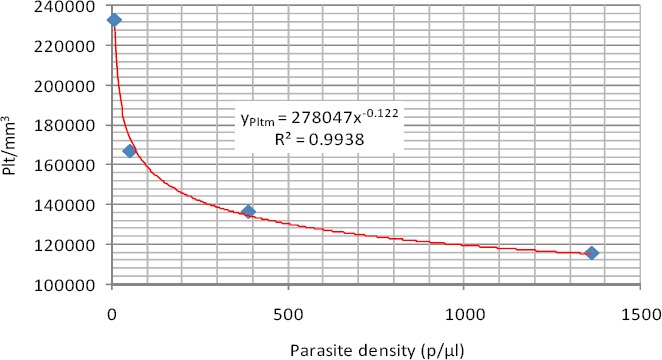
Power relationship between platelet count and parasite density (Male)

As shown Figure 7, platelet count in malaria infected male subjects exhibited a power relationship with parasite density and a very high coefficient of determination (R^2^=0.9938). The relationship is expressed in equations 9 where y_Pltm_ is the platelet count in males.

y_Pltm_ = 278047x^-0.122^ (9)

## 4. Discussion

The mean neutrophil counts in most of the infected cases studied were within the normal range of 40-75%. This finding is similar to the works of Layla et al. (2002) who reported that neutrophil count was within the normal range in most malaria patients studied in Saudi Arabia. The best predictive regression models for neutrophil count and parasite density in the studied group was negative exponential. As the neutrophil increases, the parasitemia will tend to decrease in double, triple and quadruple manner.

The inverse relationship between lymphocyte count and parasite density from this study suggests an increased apoptosis at higher parasitemia. The best predictive regression models for lymphocyte count and parasite density was logarithmic. This logarithmic relationship means that each doubling of lymphocyte count will cause the same amount of decrease in parasitemia.

The trend of decreasing platelet count with increasing levels of parsitemia observed in this study has been previously noted for *P*. *falciparum* (Perrin et al., 1982; Rojanasthien et al., 1992). The best fitting regression equation models for the relationship between platelet and parasite density for the infected male subjects were power relationships.

The empirical relationship between the parasite density in the male subjects and their mean monocyte counts were expressed by logarithmic regression model. This means that each doubling of monocyte count will cause the same amount of increase in parasitemia.

Mean eosinophil counts in the studied group showed a consistent significant increase with increasing parasite density. This agrees with earlier works by Abdallah (1988) and Jandl (1996). The empirical relationship between the parasite density and mean eosinophil counts in the studied group were expressed by logarithmic regression model. This means that each doubling of eosinophil count will cause the same amount of increase in parasitemia.

Only 5.88% of the infected males showed leucocytosis. No specific diagnostic indications are given by the white blood cell count during malaria attack and the few cases of leukocytosis may reflect the presence of concomitant bacterial infections. This is in agreement with previous work done by Layla et al. (2002), but Price et al. (2001) reported instead, a decrease in WBC count among Thai malaria patients. The empirical relationship between the parasite density and the mean WBC count was expressed by a linear regression model among the infected males. This means that as the parasite density is increasing that there is a corresponding increase in total white cell count.

Mean Hb concentration and PCV in the studied group showed a consistent decrease across the quartiles of parasite density. This observation agrees with the report of Oseni et al. (2006) that there is a steady fall in haemoglobin and PCV levels during a malaria infection. Anaemia was recorded mostly in the hyperparasitemic cases. The best fitting regression equation models for both Hb concentration and PCV and parasite density in the studied group is linear regression model. This means that as the parasite density is increasing that there is a corresponding decrease in haemoglobin concentration and PCV.

There was no significant variation between the mean values of MCHC in the studied group from the normal range of 32-36. This suggests probably that the Hb content of the circulating non-infected erythrocytes remained intact. The empirical relationship between the parasite density and the mean MCHC was expressed by a linear regression model in the studied group.

The trend of decreasing platelet count with increasing levels of parsitemia observed in this study has been previously noted for P. *falciparum* (Perrin et al., 1982; Rojanasthien et al., 1992; Sharma et al., 1992). The best fitting regression equation models for the relationship between platelet count and parasite density in the studied group is power regression model. The value of the power relationship is less than 1 (0.112), it therefore means that a unit increment in platelet count will lower the parasite density by about a square of 0.112.

## 5. Conclusion

Malaria parasitemia has been shown to have effects on some haematological parameters from this study while some haematological parameters are more predictive of malaria infection than others. Eosinophilia, thrombocytopenia and lymphopenia were identified as the key haematological indicators of malaria infection in the studied population. Eosinophilia can be used to predict the intensity of malaria infection. Thrombocytopenia is strongly associated with malaria infection and malaria parasite density. Lymphopenia is however more indicative of hyperparasitemia. Decrease in Hb and PCV in children is strongly suggestive of malaria infection. Neutrophil, monocyte and basophil counts do not have any significant relation with malaria parasitemia and are therefore not reliable indices for predicting malaria infection.

Experiential equation models have been developed from this study for estimating the values of parasite densities from haematological parameters. This could be very useful in areas where there may not be functional microscopes or competent microscopists and in medical emergencies.

## List of Abbreviations

y_nm_ neutrophil count in malaria infected males

y_lm_ lymphocyte count in malaria infected males

y_mm_ monocyte count in malaria infected males

y_em_ eosinophil count in malaria infected males

y_WBCm_ total white cell count in malaria infected males

y_Hbm_ haemoglobin concentration in malaria infected males

y_PCVm_ packed cell volume in malaria infected males

y_MCHCm_ mean cell haemoglobin concentration in malaria infected males

y_Pltm_ platelet count in malaria infected males
